# Quality of life of adult retinoblastoma survivors in the Netherlands

**DOI:** 10.1186/1477-7525-5-30

**Published:** 2007-06-04

**Authors:** Jennifer van Dijk, Saskia M Imhof, Annette C Moll, Peter J Ringens, Peggy T Cohen-Kettenis, Frank Rijmen, Jaap Huisman

**Affiliations:** 1 Department of Medical Psychology, VU University Medical Center Amsterdam, The Netherlands; 2 Department of Ophthalmology, VU University Medical Center Amsterdam, The Netherlands; 3 Department of Clinical Epidemiology and Biostatistics, VU University Medical Center Amsterdam, The Netherlands

## Abstract

**Background:**

To assess the quality of life (QoL) and predictors thereof in Dutch adult hereditary and non-hereditary retinoblastoma (RB) survivors.

**Methods:**

In this population-based cross-sectional study, a generic QoL questionnaire (SF-36) and a disease-specific interview were administered to 87 adult RB survivors aged 18 to 35 years. Their QoL data were compared with those of a Dutch healthy reference group. Among the RB hereditary/non-hereditary survivors, the QoL was compared and predictors for QoL were identified by linear multiple regression analyses.

**Results:**

As a group, RB survivors scored significantly lower than the reference group on the SF-36 subscale 'mental health' (t = -27, df = 86, p < 0.01). Hereditary RB survivors scored lower on the subscale 'general health' (t = 2.6, df = 85, p < 0.01) than non-hereditary RB survivors. Having experienced bullying, as a child was a predictor for the SF-36 subscales: 'physical functioning' (p < 0.05), 'role functioning physical' (p < 0.01), 'role functioning emotional' (p < 0.05) and 'social functioning' (p < 0.01). Having experienced bullying (p < 0.01), but also subjective experience of impairment related to RB (p < 0.05), was predictors for 'general health'. Subjective experience of impairment was a predictor for 'vitality' (p < 0.01) and 'bodily pain' (p < 0.01).

**Conclusion:**

In this exploratory study, it appears that the group of adult RB survivors experience a relatively good overall but slightly decreased QoL compared with the reference group. However, they report more problems with regard to their mental health (anxiety, feelings of depression, and loss of control). Hereditary RB survivors differ significantly from non-hereditary RB survivors only in 'general health'. Bullying in childhood and subjective experience of impairment are the main predictors of a worse QoL. In order to prevent worsening of QoL, or perhaps to improve it, clinicians should make an inventory of these issues at an early stage. We recommend further research to assess the specific psychological factors that may lead to mental health problems in this population.

## Background

Retinoblastoma (RB) is the most common malignant intraocular tumor in childhood. In the Netherlands, the incidence is 1:17,000 newborns (approximately 10–15 new patients every year) [1]. RB is generally classified into a hereditary and a non-hereditary form. The hereditary cases (40%) are caused by a germline mutation and both eyes are usually affected. In most cases (60%), the disease is non-hereditary and affects only one eye. However, it is estimated that approximately 10% of patients with unilateral RB still have a germline mutation [2]. The aim of the treatment of RB is to cure the disease and preserve vision [3]. In the western world RB has an excellent 5-year survival rate of more than 90% [4]. However, the late effects of RB (such as risk of offspring with hereditary RB, enucleation of the eye, cosmetic deformities as a result of treatment [5], enhanced risk for second primary tumors in hereditary patients [6,7] and visual impairment [8,9]) may affect many aspects of a person's life.

Measures of quality of life (QoL) enable to assess a patient's perception of the impact of their disease on their social, mental and physical state. As the vast majority (90%) of children survive RB, knowledge about their QoL is important. Although many studies have explored the effect of an ocular tumor on QoL in adults, to date only one study has assessed the long-term consequences of RB. Byrne et al. (1995) [10] concluded that self-perception of health, types of employment and life achievements did not differ between adult survivors and controls, but survivors were less likely to marry and more likely to divorce; in addition, absence of pregnancies was more common among married survivors than among controls. In childhood the QoL of RB survivors appears to be diminished, but later consequences into adulthood are not yet clear [11-13].

A study on uveal melanoma patients indicated that radiotherapy caused reduced QoL because of vision loss, pain, uncertainty about the disease, fear of recurrent tumor and death by metastatic disease [14]. However, because uveal melanoma develops later in life and has a poorer survival rate (< 50%), this condition may not be entirely comparable to the consequences experienced by adult RB patients. Studies have indicated that childhood cancer survivors experience a diminished QoL in adulthood [15-17]. It is unclear, however, whether the same applies to RB survivors. Clinicians involved in adult RB care in the Netherlands observe an extensive and far-reaching burden of that condition, even though the disease originates from early childhood. However, there are no studies on the QoL of adult RB survivors to confirm this impression, and we have found no study that elucidates the relationship between treatment for RB and long-term functioning and wellbeing.

To address this issue, we assessed QoL in Dutch adult RB survivors (aged 18–35 years) with the aim to identify predictors for a decreased QoL. A comparison was made with QoL norm data from an age-matched population of Dutch healthy persons and a comparison was made between hereditary RB survivors and non-hereditary RB survivors. We hypothesize that hereditary RB survivors have poorer QoL than non-hereditary RB survivors, considering the fact that hereditary RB survivors mostly have bilateral RB, enhanced risk of second primary tumors and risk of offspring with RB. The results of this study provide insight into the QoL of hereditary and non-hereditary RB patients that may contribute to the development of more specific psychosocial patient care.

## Methods

The present study has a cross-sectional design. From June 2005 to June 2006 all eligible RB survivors known in the national Dutch RB register [18] were invited to participate in this study. The national Dutch RB register is unique because it has maintained virtually complete data from 1945 until 2006. Eligibility requirements for inclusion in this study were: (1) age between 18–35 years, (2) sufficient command of the Dutch language to understand the questionnaire and the interview; (3) adequate cognitive abilities for the same reason, and (4) treatment for RB in the VU University Medical Center (Amsterdam), the University Medical Center Utrecht, or the University Medical Center St. Radboud (Nijmegen); these three hospitals have had treatment responsibility for 86% of the national patient population.

Survivors of RB were sent a letter with an invitation to participate. Informed written consent was obtained from all respondents. Participants who had agreed to participate were contacted by telephone, information was given, and appointments were made to visit the survivors at home for personal communication and a semi-structured interview. One week before the home visit, participants received the SF-36 self-report questionnaire to be filled out. This study was approved by the Ethics Committees, and was conducted in accordance with the principles of the Helsinki declaration.

### Measures

#### Hospital charts

Predictors of QoL that were obtained from hospital charts were: age at diagnosis, date of birth, gender (male/female), hereditary status (non-hereditary/hereditary), laterality (unilateral/bilateral), type of treatment, and visual acuity. Treatment was categorized as: 1) only enucleation, 2) only external beam radiotherapy, 3) combination of enucleation and radiotherapy, and 4) a combination of enucleation and remaining therapies (chemothermotherapy, plaque radiotherapy, laser photocoagulation and cryotherapy) [2]. Visual acuity was defined as the visual acuity after subjective refraction in the participant's better eye and categorized according to the WHO guidelines [19] as: 1) normal vision (> 0.3), 2) low vision (0.05–0.3), and 3) blindness (< 0.05). If hospital charts were not available the information was obtained by personal communication.

#### Semi-structured interview

Prior to the home visits, the semi-structured interview was developed. Topics for the interview were obtained from literature, clinical observations and from focus group discussions [20] with eight experts and six RB survivors. Extensive semi-structured interviews were conducted (JvD) with the RB survivors focusing on early adaptation to the diagnosis, and perceived burden of their illness in relation to educational achievement and social functioning. For the present study, we only report data from the semi-structured interviews that are appropriate for quantification. Educational level was categorized as: 1) lower (primary or secondary school), 2) middle (high school or professional education), and 3) higher (college and university). Content analysis was used to extract data on perceived impairment related to RB (yes/no), and on the experience of being bullied because of RB (yes/no). Furthermore, data on marital status (single/living together) and life events were extracted. The life events were inventoried by asking whether any of the following life events had occurred: accidents, other disease besides RB, disease of a member of the family, death of a family member or death of a best friend, divorce, divorce of parents, moving, sexual abuse, enprisonment, enprisonment of parent, loss of job, admission into a psychiatric hospital, admission of a parent into a psychiatric hospital. Answers were categorized into: 1) no life events occurred, 2) one or two life events occurred, 3) more than two life events occurred.

#### Quality of life measures

The dependent variable, QoL, was measured by the Dutch version of the Medical Outcome Study Short-Form 36 (SF-36) [21]. The SF-36 is a widely used and well-validated generic QoL instrument. Recently, the SF-36 was recommended when used in studies in long-term survivors of childhood cancer [22]. The SF-36 contains eight separate subscales representing physical, psychological, and social functioning. It measures four dimensions of physical health: 'physical functioning' (ability to perform physical activities without limitations), 'role functioning physical' (possibility to work or perform daily role functions without interference from physical health problems), 'bodily pain' (pain interfering with daily activities) and 'general health perception' (self-evaluation of overall health status) and four dimensions of mental health: 'vitality' (energy), 'social functioning' (impact of physical or emotional problems on normal social activities), 'role functioning emotional' (capacity to perform daily activities without interference from emotional problems) and 'mental health' (anxiety, feelings of depression and loss of control). Per domain, raw scores were transformed to standardized scores on a scale from 0 to 100, with a higher score reflecting a better QoL. In order to compare the QoL of RB survivors with the general Dutch population, SF-36 scores available from age-matched controls of the general Dutch population were used [23]. The proportion females in the age-matched general Dutch population is 53% and is not statistically significant (Chi-Square Test, p = 0.26) from the proportion females among the RB survivors (61%). Internal consistency reliability (Cronbach's alpha) for the different areas of physical and mental health range from 0.78 ('general health') to 0.92 ('physical functioning') [23].

### Statistical analysis

Analyses were carried out using the software package SPSS 11.5 for Windows. Differences in socio-demographic and psychosocial characteristics between hereditary and non-hereditary RB survivors were examined with Student's t-tests. Differences in frequencies were examined with Chi-Square tests. One sample t-tests were used to test differences in SF-36 subscales between the RB group and the reference group. Independent sample t-tests were used to compare mean scores of hereditary RB survivors with non-hereditary RB survivors, because we expect that hereditary RB survivors have poorer QoL than non-hereditary RB survivors. Possible predictors of the QoL subscales were studied by linear multiple regression. Stepwise, univariate general linear model analysis was used to determine the best predictive model of each SF-36 subscale score (dependent variables). Variables that were likely to affect the SF-36 subscales were included in the regression model as a fixed set of variables. These (independent) variables were: gender, life events, socio-economic status, age at interview, age at diagnosis, type of treatment, heredity, laterality, visual acuity, subjective experience of impairment, and experience of being bullied in childhood. Variables that were likely to affect the QoL subscales with a significant effect (p < 0.05) were included in the final model and reported. In all tests, p-values of less than 0.05 were deemed to be statistically significant.

## Results

Of the 148 adult survivors that were eligible for our study, 21 (14%) survivors could not be traced and were therefore not approached for participation. Of the remaining 127 survivors, 95 (75%) survivors agreed to cooperate and 87 (69%) of them completed the study, including the SF-36 questionnaire and a semi-structured interview. Of the non-participating 32 survivors (25%): 20 (16%) preferred not to participate, 10 (8%) did not respond within the study period, and 2 (2%) had moved abroad. Reasons for not taking part were mainly lack of time, no interest in the study or not wanting to be confronted again with their disease. Comparison of age, hereditary status, and treatment revealed no significant differences between the participating RB survivors and the non-participants. However, more women (61%) than men were willing to participate in the study.

Table 1 presents data on socio-demographic and psychosocial characteristics of the study participants, and differences between hereditary and non-hereditary subgroups of RB survivors.

**Table 1:** Socio-demographic and psychosocial characteristics

	All RB survivors (N = 87)	Hereditary RB survivors (N = 36)	Non-hereditary RB survivors (N = 51)
Age at interview *Years: mean (SD)*	26.4 (5.3)	26.8 (4.8)	26.2 (5.7)
			
Age at diagnosis *Years: mean (SD)*	2 (2.0)	0.69 (1.6)*	2.7 (1.8)*
			
Gender (N (%))			
*Female*	53 (61%)	25 (69%)	23 (45%)
			
Education (N (%))			
*Low*	16 (18%)	8 (22%)	8 (16%)
*Middle*	33 (38%)	14 (39%)	19 (37%)
*High*	38 (44%)	14 (39%)	24 (47%)
			
Marital status (N (%))			
*Single*	37 (43%)	15 (42%)	21 (41%)
			
Laterality (N (%))			
*Unilateral rb*	56 (64%)	5 (14%)*	51 (100%)*
			
Treatment (N (%))			
*Only enucleation*	48 (55%)	5 (14%)*	43 (84%)*
*Only radiotherapy*	14 (16%)	14 (39%)*	
*Combi enucleation + radiotherapy*	21 (24%)	14 (39%)*	7 (14%)*
*Combi enucleation + chemo/laser*	4 (5%)	3 (8%)*	1 (2%)*
			
Visual acuity (N (%))			
*Normal vision*	78 (90%)	27 (75%)*	51 (100%)*
*Low vision*	6 (7%)	6 (17%)*	
*Blindness*	3 (3%)	3 (8%)*	
			
Life events (N (%))			
*None*	20 (23%)	8 (22%)	12 (24%)
*One or two*	42 (36%)	17(47%)	25 (49%)
*More than 2*	22 (25%)	9 (25%)	13 (26%)
*Missing*	3 (3%)	2 (6%)	1 (2%)
			
Being bullied (N (%))			
*Yes*	77 (88%)	34 (94%)	43 (84%)
			
Perceived impairment (N(%))			
*Yes*	48 (55%)	20 (56%)	28 (55%)

*Significant difference (p < 0.05) between hereditary and non-hereditary survivors of RB.

### Quality of life (SF-36)

Table 2 shows standardized scores derived from the SF-36 with the comparison between the RB group and the Dutch reference group. Survivors of RB scored lower on the subscale mental health (mean difference = 18.1, p < 0.001) compared with the norm data from an age-matched Dutch healthy reference group [23]. All other SF-36 subscales showed no significant differences between the RB group and the reference group.

**Table 2:** Comparison of standardized SF-36 scores (SD) between RB group and Dutch reference group, and between non-hereditary RB survivors and hereditary RB survivors

SF-36 Domains	Dutch reference group (N = 701)	RB group (N = 87)	Hereditary RB survivors (N = 36)	Non-hereditary RB survivors (N = 51)
Physical functioning	93.1 (11.8)	93.2 (12.9)	91.9 (15.9)	94.5 (9.9)
Role functioning physical	86.4 (27.6)	85.3 (29.7)	78.5 (32.8)	90.0 (26.7)
Bodily pain	80.9 (19.4)	84.5 (20.5)	82.9 (20.6)	85.3 (20.6)
General health	78.2 (17.3)	78.6 (20.4)	72.0 (21.6) *	83.4 (18.5) *
Vitality	70.7 (16.4)	71.5 (17.1)	72.2 (18.2)	70.9 (16.5)
Social functioning	87.8 (19.1)	89.2 (18.0)	88.5 (16.5)	89.5 (19.3)
Role functioning emotional	85.4 (30.0)	88.1 (28.3)	88.9 (27.6)	87.3 (29.3)
Mental health	78.7 (15.2) *	60.6 (6.3) *	62.1 (7.1)	59.5 (5.6)

* Significant difference between groups (p < 0.01)

Table 2 shows standardized scores derived from the SF-36 with the comparison between hereditary RB survivors vs non-hereditary RB survivors. Hereditary RB survivors scored lower on the subscale general health (mean difference = 8.6, p = 0.01) compared with the non-hereditary RB survivors. All other SF-36 subscales showed no significant differences between the hereditary RB survivors and non-hereditary RB survivors.

### Predictors of quality of life

The results of the multiple regression analyses can be found in Table 3. Eleven variables were included as independent predictors in a linear multiple regression model. Having experienced bullying was an independent predictor of: physical functioning (p = 0.04), role functioning physical (p = 0.002), role functioning emotional (p = 0.049) and social functioning (p = 0.011). Having experienced bullying (R^2 ^= 0.067, p = 0.023) and perceived impairment (R^2 ^= 0.059, p = 0.033) were both predictors of general health. Perceived impairment was a predictor of vitality (p < 0.001) and bodily pain (p = 0.039). Age, gender, marital status, educational level, life events, heredity, type of treatment, visual acuity and laterality did not predict any of the QoL aspects of the SF-36.

**Table 3:** Multiple regression analyses (method stepwise)

Dependent variable	F	p	R^2^	Independent variable	β (standardized)
Physical functioning	4.258	0.042	0.049	Bullying	-0.222
Role-physical functioning	9.812	0.002	0.107	Bullying	-0.327
Role-emotional functioning	4.000	0.049	0.047	Bullying	-0.216
Social functioning	6.703	0.011	0.076	Bullying	-0.275
General health	4.998	0.003	0.167	Bullying Impairment	-0.293 -0.233
Vitality	14.364	0.000	0.156	Impairment	-0.394
Bodily Pain	4.431	0.039	0.054	Impairment	-0.232

## Discussion

The present study assessed the QoL of a unique Dutch population of RB survivors using the SF-36 questionnaire and a semi-structured interview focusing on early adaptation to the diagnosis and the perceived burden of their illness in relation to educational achievement and social functioning. To our knowledge, this is the first study to examine long-term QoL in adult RB survivors.

Our results show no significant differences between the adult RB survivors compared to the healthy reference group in the QoL measures, except for the mental health scale. RB survivors learn to live with many of the consequences of their disease, but reported more problems with regard to their mental health compared with the reference group. This result is clinically significant, because the mean MH score in the RB group was 18.1 points (3 standard deviations (SD), p < 0.001) lower than that of the Dutch reference group. A difference of 18.1 points on a scale from 0–100, means almost 20% difference between the RB survivors and the Dutch reference group on the MH scale. In particular, anxiety, feelings of depression and loss of control seem to have a negative impact on their lives. These unfavorable mood states might be caused by their feelings of being different from others. According to our survivor's reactions during the interview, this often originates from having been bullied about their facial appearance or prosthesis, and/or their visual impairment or blindness. Besides that, loss of control may lead to feelings of depression. In particular, realization about their loss of control appears to be connected to the emotion of shame [24], and experiencing shame in childhood can be a forerunner of depression [25]. In the present study, our group of RB survivors are more anxious and worried compared with the reference group. They grew up with uncertainty about their facial appearance and the feeling of being different; this can lead to feelings of shame, which may influence their perception and experiences during general development and may eventually result in depression. This finding is consistent with results from other diseases with atypical visible facial characteristics, such as strabismus and patients with a cleft. Strabismus patients experience more social anxiety [26], and have more difficulties with self-image and interpersonal relationships [27] in comparison with the reference group. Persons with visible facial characteristics (like a cleft), expressed greater dissatisfaction with their appearance [28]. Further research should be conducted to identify the specific psychological factors that lead to problems in mental health in this population.

The second aim of this study was to compare the QoL of hereditary RB survivors with that of non-hereditary RB survivors. The general health perception of hereditary RB survivors was significantly impaired compared with non-hereditary RB survivors. This probably reflects a realistic view of their situation. Hereditary RB survivors often experience more physical problems than the non-hereditary group: i.e. they are often subjected to more treatments [12], are bilaterally affected, have a greater chance of visual impairment, and are at greater risk of developing second primary tumors. It is remarkable, however, that despite these additional problems they do not report to be affected in other QoL areas.

Another striking result is that non-hereditary RB survivors also experience anxiety concerning second primary tumors, even though they are at less risk for this compared with hereditary RB survivors. Nowadays, clinicians can estimate the probability of survivors passing on the disease to their offspring or the probability of developing second primary tumors. However, for patients with a rare disease, the impact of hearing that there is a 'low probability' of something occurring might be received differently from how clinicians may expect. From a healthy person's perspective, a low probability generally means a minor or no chance of having/passing on a certain disease. For a person with a rare disease, however, a low probability might logically mean the same but their own reality of having a rare disease may have proven otherwise. The discrepancy between theory and their own reality might introduce fear (or at least some existential thoughts) about why they could not escape from developing a rare disease. This might explain the fears also experienced by non-hereditary RB survivors. Therefore, clinicians should be aware that RB survivors (including non-hereditary RB) might interpret these probabilities differently from what may be expected. A similar tendency was also found among women at risk for breast cancer [29].

The third aim of the study was to gain insight into the predictors of QoL. Bullying in childhood and impairment appeared to be the major predictors of QoL of our RB survivors. According to the interview results, the reasons for bullying were in most cases related to the appearance of the eye or to the survivor's facial appearance. This was also found in another study on children with RB in which parents reported that their child had experienced bullying related to either facial appearance or the ocular prosthesis [11]. The association between bullying and QoL has also been reported in other types of childhood cancer [30].

Most of the impairments mentioned by survivors were associated with a wide range of activities related to their visual acuity. Lamoureux et al. (2004) [31] concluded that the areas of greatest restriction in people with impaired vision were associated with reading, outdoor mobility, participation in leisure activities and shopping. It is understandable that a serious restriction in these activities is negatively related to the experience of health and vitality.

This study focused on the QoL of a diverse and rare population of RB survivors and provides information on a population-based RB group. Nevertheless, a number of study limitations should be considered when interpreting the results. First, since the possibilities of treatment have improved over time, our results may no longer apply to survivors who have been treated more recently. On the other hand, some form of treatment will always be necessary and the current RB treatments still do not leave the appearance of the RB survivor totally unaffected; further research is therefore desirable.

Second, it is conceivable that some of the RB survivors who did not participate in the present study experienced a poorer QoL than those who did participate. Indeed, several non-participants refused participation because they did not want to be confronted with their disease again; if this subgroup consists of those who do not accept their disease as well as the other subgroups, then the overall QoL of RB survivors might be worse than reported here.

Third, we are aware of the fact that some SF-36 subscales show ceiling effects [22]. Therefore, it can be that the effects we found were underestimated. Estimates of the coefficients and their standard errors are robust to the non-normal distributions. Although the tests confidence intervals originate from normal distributions the consequences of violating this assumption are minor with sufficient sample size.

## Conclusion

In conclusion, our exploratory study indicates that adult RB survivors generally experience a relatively good QoL compared with the reference group. However, RB survivors in our study have more problems with regard to their mental health; particularly anxiety, feelings of depression and loss of control influence their lives negatively. These unfavorable mood states may be caused by their feelings of being different from others and by childhood bullying. Hereditary RB survivors differ from non-hereditary RB survivors, probably justifiably, only in their experience of general health. In patients with RB, decreased QoL may arise from the psychological effects of having been bullied and from the negative experience of their impairments related to the disease. In order to prevent worsening of QoL, clinicians should not hesitate to address these issues at an early stage. We recommend further research to assess the specific psychological factors that may lead to mental health problems in this population.

## Competing interests

The author(s) declare that they have no competing interests.

## Authors' contributions

JvD has coordinated the research, collected and analysed the data and drafted the manuscript. SMI, ACM and JH have participated in the design of the study, interpreted the data and revised the manuscript. PJR and PTCK interpreted the data and revised the manuscript. FR contributed to the statistical analysis and revised the manuscript. All authors read and approved the final manuscript.
